# Prediction of hamstring tendon autograft diameter using preoperative measurements with different cut-offs between genders

**DOI:** 10.1186/s40634-023-00569-0

**Published:** 2023-01-21

**Authors:** Mohammad Movahedinia, Sajjadeh Movahedinia, Seyedreza Hosseini, Ali Motevallizadeh, Bentolhoda Salehi, Babak Shekarchi, Mostafa Shahrezaee

**Affiliations:** 1 Department of Orthopedic Surgery, Aja University of Medical Sciences, Tehran, Iran; 2 Department of Pathology, School of Medicine, Tehran University of Medical Sciences, Tehran, Iran; 3 Bone Joint and Related Tissues Research Center, Akhtar Orthopedic Hospital, Tehran, Iran; 4 School of Medicine, Kerman University of Medical Sciences, Kerman, Iran; 5 Radiation Sciences research Center, Aja University of Medical Sciences, Tehran, Iran

**Keywords:** Hamstring tendon autograft, ACL reconstruction, Cross-sectional area, Sensitivity, Specificity

## Abstract

**Purpose:**

Studies have suggested some predictors for hamstring tendon (HT) autograft diameter based on anthropometric factors and preoperative magnetic resonance imaging (MRI) with variable results. Some authors have attributed the variability to gender differences. This prospective cohort reports the sensitivity and specificity of anthropometric and MRI predictors in males and females separately to determine the difference.

**Methods:**

Forty-two eligible patients who underwent anterior cruciate ligament reconstruction (ACLR) and MRI in our center were included. ACLR was performed by the senior surgeon using a 4-stranded HT autograft for all patients. A blinded musculoskeletal radiologist measured the cross-sectional area (CSA) of gracilis and semitendinosus tendons using the free-hand region of interest tool for all patients. An orthopaedic resident (PGY4) collected anthropometric factors and measured intraoperative autograft diameter.

**Results:**

Mean intraoperative autograft diameter was 8.0 mm. Females had a significantly lower autograft diameter (7.4 vs. 8.2, *P* < 0.001), smaller gracilis (6.9 vs. 7.9, *P* = 0.003) and semitendinosus CSA (11.5 vs. 12.8, *P* = 0.014) compared to males. ROC curve analysis resulted different cut-off values with high sensitivity and specificity for semitendinosus and combined CSA regarding gender.

**Conclusion:**

Based on the results of this study, CSA of either isolated or combined HTs on preoperative axial MRI, height, and weight are the strongest predictors of intraoperative autograft diameter. It is suggested to consider different cut-offs for males and females to have a better clinical guide for surgeons.

**Level of evidence:**

Level II.

## Background

Hamstring tendon (HT) as an autograft for anterior cruciate ligament reconstruction (ACLR) has received considerable attention because of its good clinical and biomechanical results and low donor site morbidity [23, 24]. Studies have shown that the risk of autograft rupture increases with autograft diameters less than 8 mm, [5, 14] that may force the surgeon to use other autografts or even allografts. This leads to increased operation time and risk of infection, further manipulation of the patient’s soft tissue, and probably lower outcomes [23]. Finding parameters based on which the diameter of the autograft can be predicted before surgery is the key to solving this problem [25].

Studies have shown a correlation between the diameter of tendons on preoperative magnetic resonance imaging (MRI) and the diameter of the autograft harvested during surgery [6, 12, 22]. According to the latest systematic review in this field, this correlation has been mostly reported as high for quadriceps tendons and bone-patellar tendon-bone autografts, but moderate for the HT [1]. Therefore, the efficacy of MRI measurement has been left indistinctive as a predictor for adequate HT autografts during ACLR.

There are also studies that have investigated the role of anthropometric variables such as sex, height, and weight in predicting autograft diameter [15, 20, 21]. Contrary to the highly reliable results of MRI-based studies, the results of these studies have not reached consistent conclusions. Therefore, despite the variability observed in studies, MRI still seems to be the best predictor of HT autograft diameter during ACL [1].

Many studies have attributed the variability of anthropometric factors in predicting the intraoperative autograft diameter to gender differences [2, 4, 17, 21, 25]. We hypothesized that assessing the MRI measurements separately in males and females can provide a more accurate prediction of the autograft diameter. Hence, this prospective study was designed to evaluate the predictability of HT autograft diameter using anthropometric factors and preoperative MRI measurements separately in males and females.

## Material and methods

After receiving Institutional Review Board (IRB: IR.AJAUMS.REC.1400.196) approval and written informed consent, 42 patients were included in the study and underwent ACLR from February 2021 to July 2022 using a 4-stranded HT autograft. The inclusion criteria included patients older than 18 years who were candidates for ACLR based on clinical findings and MRI. Exclusion criteria included: Previous surgery on the same knee or the contralateral lower limb, multiligament injuries, and a history of hamstring injury. Patients who underwent MRI at another center were also excluded from the study. All patients were operated on by a senior surgeon in the same orthopaedic hospital. The MRI of all patients was performed at most 1 month before surgery using the same 3 T scanner (Siemens, Erlangen, Germany) and a specific knee coil.

**Fig. 1:**
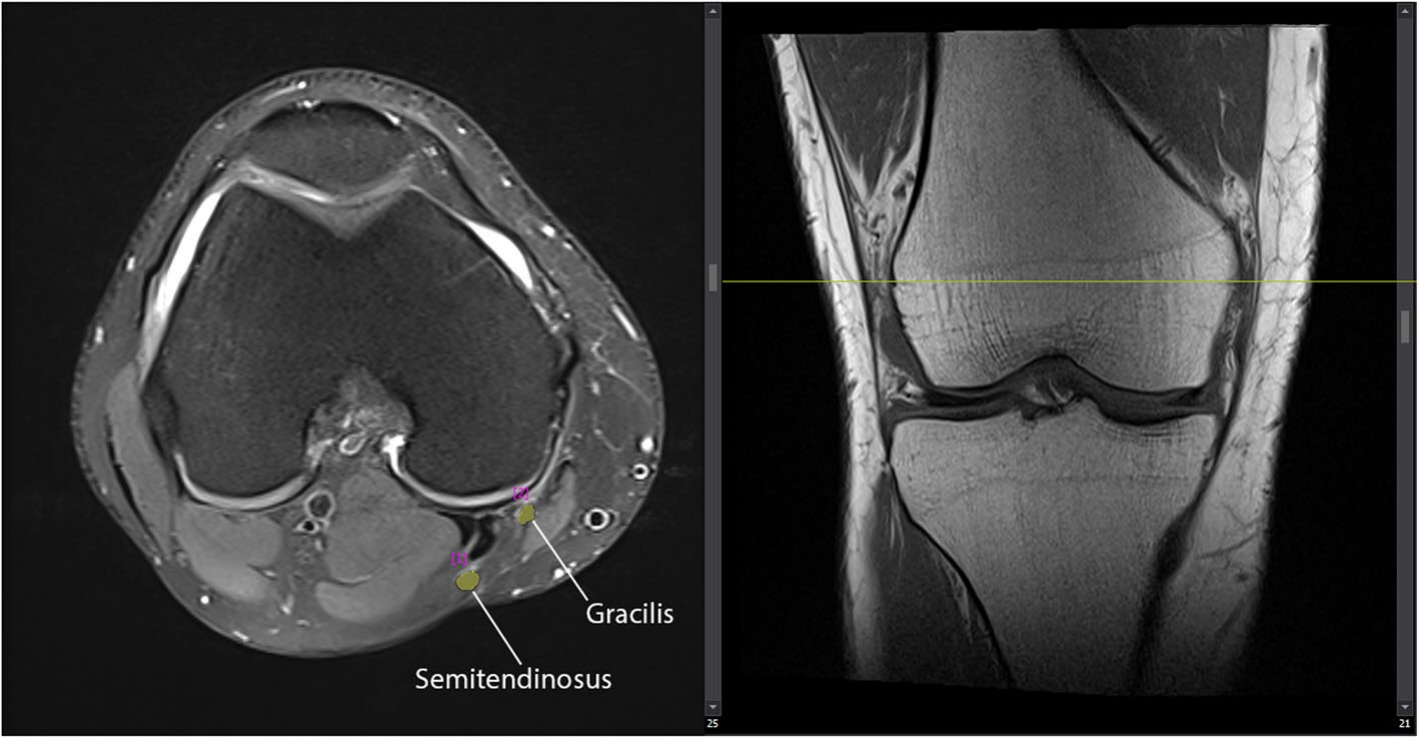
Coronal sequence (right) was used to indicate the medial epicondyle of the distal femur as a reference where the CSA was measured on axial sequences (left)

**Fig. 2:**
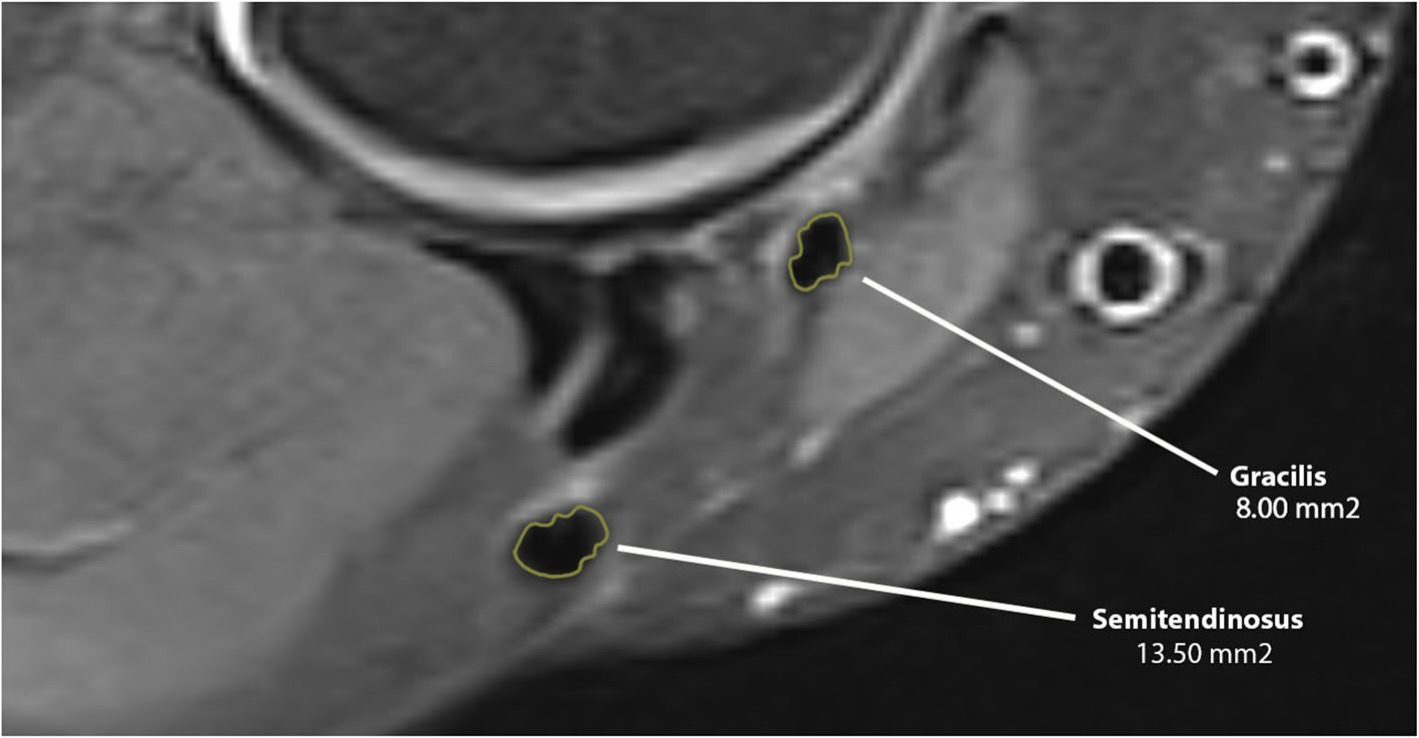
The CSA measurement for semitendinosus and gracilis was performed at 2 x magnification

Anthropometric factors were recorded during the week before surgery by an orthopaedic resident (PGY4) and included sex, age, height, weight, and body mass index (BMI). A musculoskeletal radiologist measured the cross-sectional area (CSA) of gracilis and semitendinosus tendons for all patients. Using the coronal sequence as a cross reference, he measured CSA on axial sequences at the level of the medial epicondyle of the distal femur since it has been proven to be the best location for CSA measurement of HTs [11] (Fig. 1). CSA measurements were made by the free-hand region of interest tool available in the PACS system at two-times magnification, using the same method for all patients (Fig. 2). The radiologist was blinded to the intraoperative measurements until the end of the study.

### Surgical technique

Both gracilis and semitendinosus tendons were harvested using the same method by the senior surgeon in all patients. A 3–4 cm oblique incision was placed medial to the tibial tuberosity, the sartorial fascia was incised, and each HT was isolated after the removal of excess fascial bands. After separating from the insertion site, gracilis and semitendinosus tendons were harvested by a closed-loop tendon stripper. Then the tendons were folded over to form a 4-stranded autograft. The diameter of the final HT autograft was determined using the sizing cylinder. All grafts were prepared and measured by the same orthopaedic resident (PGY4) under the supervision of the senior surgeon. Autograft measurement was performed when the routine diagnostic arthroscopy was finished and just before the autograft fixation for all patients, as Cruz et al. [7] found that grafts may vary in size as much as 1 mm when measured at different times of surgery. Finally, femoral and tibial sides were fixed using the Endobutton and Biosure screw, respectively.

### Statistical analysis

SPSS version 19 (IBM; Armonk, NY, USA) software was used for data analysis. Mann-Whitney U test, and t-test were carried out for comparison of quantitative variables between groups and the Chi-square test was used for qualitative variables. The normality of the distribution of the quantitative data was investigated by the Kolmogorov-Smirnov statistic. Continuous variables were presented with mean ± standard deviation (SD). Spearman correlation coefficient was used to investigate the relationship between intraoperative autograft diameter and preoperative predictors. Receiver operating characteristic (ROC) analysis was performed to calculate the cut-off point in CSA measurement to reach an autograft thicker than 8 mm intraoperatively. A two-sided P-value of less than 0.05 was reported as statistically significant. Multiple regression analysis was done with selected preoperative predictors. We assessed the sensitivity and specificity of CSA measurement of MRI at the calculated cut-offs to predict adequate autograft (≥8 mm).

## Results

Of the 42 eligible patients participating in this study 32 (76.2%) were male. The mean age of the patients was 32.8 years, ranging from 19 to 40 years. The mean interval between preoperative MRI and anthropometric measurements and ACLR was 18 (4–26) and 3 (1–6) days, respectively. The mean intraoperative HT autograft diameter was 8.0 mm. In 27 patients (64.3%), an HT autograft diameter of 8 mm or more was harvested during surgery. Anthropometric factors and preoperative measurements are reported in Table 1. None of the patients required alternative autograft or allograft according to intraoperative assessments.

**Table 1:** Anthropometric factors and preoperative MRI measurements of study participants

Variable	Mean ± SD	Min	Max
**Age, y**	32.8 ± 5.1	19	40
**Height, cm**	173.8 ± 5.6	164	185
**Weight, kg**	77.1 ± 7.3	64	95
**BMI, kg/m**^**2**^	25.4 ± 2.0	21.2	29.6
**Graft Diameter, mm**	8.0 ± 0.6	7.0	9.0
**ST CSA, mm**^**2**^	12.5 ± 1.5	9	15
**Gr CSA, mm**^**2**^	7.6 ± 1.0	6	9
**ST + Gr CSA, mm**^**2**^	20.2 ± 2.4	15	24

*STT CSA* Cross-sectional area of semitendinosus tendon, *Gr CSA* Cross-sectional area of gracilis tendon, *BMI* Body mass index

Females had a significantly lower HT autograft diameter (7.4 vs. 8.2, *P* < 0.001), smaller gracilis CSA (6.9 vs. 7.9, *P* = 0.003), and smaller semitendinosus CSA (11.5 vs. 12.8, *P* = 0.014) compared to males.

Results of Mann-Whitney U and t-test analysis showed that patients with an HT autograft diameter ≥ 8 mm, were significantly taller (*P* = 0.001) and heavier in body weight (*P* = 0.036) with a greater gracilis (*P* < 0.001), semitendinosus (*P* < 0.001), and combined CSA (*P* < 0.001). Table 2 compares anthropometric factors and preoperative MRI measurements between patients with an adequate (≥8 mm) and those with an inadequate (< 8 mm) autograft diameter.

**Table 2:** Demographics and MRI measurements compared between groups with measured HT auto autograft diameter of ≥8 mm and < 8 mm

Variable	Graft Diameter ≥ 8 mm	Graft Diameter < 8 mm	***P***-value
**Age, y**	32.5 ± 5.6	33.3 ± 4.2	0.635
**Height, cm**	175.8 ± 5.0	170.3 ± 4.3	0.001
**Weight, kg**	78.8 ± 7.9	73.9 ± 5.0	0.036
**BMI, kg/m**^**2**^	25.4 ± 2.3	25.5 ± 1.5	0.868
**ST CSA, mm**^**2**^	13.1 ± 1.4	11.4 ± 1.1	< 0.001
**Gr CSA, mm**^**2**^	8.1 ± 0.8	6.9 ± 0.7	< 0.001
**ST + Gr CSA, mm**^**2**^	21.3 ± 2.1	18.3 ± 1.5	< 0.001

*STT CSA* Cross-sectional area of semitendinosus tendon, *Gr CSA* Cross-sectional area of gracilis tendon, *BMI* Body mass index

Bivariate correlation analysis between HT autograft diameter and independent variables revealed that height, weight, semitendinosus CSA, gracilis CSA, and combined CSA, positively correlated with HT autograft diameter harvested during surgery (Table 3).

**Table 3:** Bivariate correlation analysis between HT autograft diameter and anthropometric and MRI measurements

Variables	Spearman correlation coefficient	***P***- value
**Age, y**	−0.10	0.52
**Height, cm**	0.63	< 0.001**
**Weight, kg**	0.32	0.04*
**BMI, kg/m**^**2**^	−0.17	0.29
**ST CSA, mm**^**2**^	0.68	< 0.001**
**Gr CSA, mm**^**2**^	0.79	< 0.001**
**ST + Gr CSA, mm**^**2**^	0.74	< 0.001**

*STT CSA* Cross-sectional area of the Semitendinosus tendon, *Gr CSA* Cross-sectional area of the Gracilis tendon, *BMI* Body mass index

*Correlation Significant at 0.05 level; **Correlation Significant at 0.01 level

For adequate HT autograft diameter (≥8 mm) prediction, ROC curve analysis determined a cut-off value of 13.5mm^2^ for semitendinosus CSA with a sensitivity of 40.7% and a specificity of 93.3%, 7.5mm^2^ for gracilis CSA with a sensitivity of 88.9% and a specificity of 80.0%, and 20.5mm^2^ for combined ST + Gr CSA with a sensitivity of 70.4% and a specificity of 93.3%. Considering the significant different anthropometric factors between genders, analysis was performed for each sex separately, as demonstrated in Table 4.

**Table 4:** Calculated cut-offs for preoperative predictors of autograft diameter according to sex

	Overall			Males			Females		
	Cut-off	SN	SP	Cut-off	SN	SP	Cut-off	SN	SP
**ST CSA, mm**^**2**^	13.5	40.7%	93.3%	13.5	45.8%	87.5%	11.5	100%	71.4%
**Gr CSA, mm**^**2**^	7.5	88.9%	80%	7.5	91.7%	62.5%	7.5	66.7%	100%
**ST + Gr CSA, mm**^**2**^	20.5	70.4%	93.3%	20.5	75%	87.5%	19.5	66.7%	100%

*STT CSA* Cross-sectional area of semitendinosus tendon, *Gr CSA* Cross-sectional area of gracilis tendon, *SN* Sensitivity, *SP* Specificity

## Discussion

Based on the findings of this study, to make the use of preoperative MRI measurements more practical, it is better to assess the predictors in males and females separately. We found height (*r* = 0.63), weight (*r* = 0.32), and the CSA of isolated and combined HTs, especially gracilis (*r* = 0.79) as the best predictors for the HT autograft diameter are. According to our results, a minimum gracilis CSA of 7.5mm^2^ at the level of the medial epicondyle on axial MRI can assure surgeons of an intraoperative autograft ≥8 mm with 88.9% sensitivity and 80% specificity. Interestingly, when the analysis was performed for males and females separately, the sensitivity increased to 91.7% for males and the specificity to 100% for females at the same cut-off. Regarding semitendinosus or combined HTs CSA measurements, a lower cut-off was obtained for females. This is in line with the hypothesis of this study, which states to have a more practical prediction of the HT autograft diameter, different cut-offs should be considered in males and females (Table 4). From a clinical point of view, we found that females with adequate HT autograft diameter may have less preoperative CSA measurements compared to males. Among all the anthropometric factors investigated in this study, the HT autograft diameter was significantly correlated with height and weight. This is consistent with previous studies showing height and weight as the strongest predictors of autograft diameter among anthropometric factors [8, 15, 18–20]. We found a higher degree positive correlation between gracilis CSA and HT autograft diameter than height (*r* = 0.79 vs. 0.63). A multiple linear regression model showed that gracilis CSA (*P* < 0.001) and height (*P* = 0.004) are statistically important determinants of HT autograft diameter with the following linear regression equation:

HT autograft diameter = 0.373 × Gr CSA (mm^2^) + 0.037× Height (cm) - 1.35.

Few studies have evaluated both MRI and anthropometric variables as predictors of HT autograft diameter simultaneously [6, 9, 10, 13, 22]. One important drawback of these studies is that the patients underwent imaging using a combination of 1.5 T and 3 T MRI. This is while 1.5 T and 3 T MRI can work with different accuracies in predicting the autograft diameter [12]. However, L. Thwin et al. [22] revealed no significant difference between 1.5 T and 3 T MRIs. In the current study, all patients underwent imaging using the same 3 T MRI. Hollnagel et al. [12] showed that CSA measurement of semitendinosus at the level of medial femoral condyle using 3 T MRI is the best correlated with intraoperative autograft diameter.

In the study of Mr. Grawe et al., [9] although anthropometric factors and CSA measurements were investigated simultaneously, the wide range of patients in terms of age (9 to 58 years old) was a serious confounder. While the authors did not consider analysis or a way to adjust this confounding effect. This confounding effect stems from the hypothesis that adolescents typically have smaller anthropometric factors. All patients evaluated in this study were older than 18 years. Leiter et al. [13] used specific computer software to measure CSA that is not available everywhere. We used a simple free-hand region of interest tool that is available in every PACS system and approved by the US Food and Drug Administration [3].

The study by Leiter et al. [13] and Corey et al. [6] were done retrospectively, which questions the accuracy of the measurements especially since the height and weight of the patients were self-reported, which can cause considerable bias in the results. Since measurements of autografts during surgery are usually estimated when they are not performed for the research sample, the retrospective nature of a study can be so limiting that no meaningful results can be obtained like the study of Oliva Moya et al. [16]. Our study was a prospective cohort in which we tried to make the preparation and measurement of the autograft, imaging measurements, and recording of anthropometric factors the same for all patients. Considering the prospective design of this study, unlike Heijboer et al., [10] we considered the time interval between the recording of MRI and anthropometric factors with surgery to be the same in all patients, which was not more than 1 month. All of these can be possible reasons for the higher degree positive correlation between predictors and autograft diameter in this study compared to the few studies that evaluated both MRI and anthropometric variables simultaneously [6, 9, 10, 13, 22].

However, this study also has shortcomings that can be controlled in other studies to obtain more reliable results. In this study, CSA was measured only at the level of the medial epicondyle, while studies [12] have shown that the correlation between CSA and autograft diameter can be different by measuring it in different areas. However, it has been proven that the best area to measure CSA is the medial condyle or epicondyle [11, 12]. Radiological measurements in this study were performed by an expert musculoskeletal radiologist. Not using different reviewers, including orthopaedic surgeons, has made it impossible to obtain intra- and interrater reliability of the measurements. This can affect the clinical applicability of the results of this study. The small study population is another important limitation of this study that implies the cut-offs and the equation reported in this study should be used with caution.

## Conclusion

Preoperative CSA of HTs, especially gracilis on MRI, length, and weight are most predictive for an adequate diameter of 4-stranded HT autograft harvested during ACLR.

## Abbreviations

CSA: Cross-sectional area
MRI: Magnetic resonance imagery
ACLR: Anterior cruciate ligament reconstruction
HT: Hamstring tendon
Gr: Gracilis
ST: Semitendinosus
